# Effectiveness of Telemedicine Solutions for the Management of Patients With Diabetes: Protocol for a Systematic Review and Meta-Analysis

**DOI:** 10.2196/22062

**Published:** 2020-11-03

**Authors:** Sisse Laursen, Stine Hangaard, Flemming Udsen, Peter Vestergaard, Ole Hejlesen

**Affiliations:** 1 Department of Health Science and Technology University of Aalborg Aalborg Øst Denmark; 2 University College Nordjylland Aalborg Øst Denmark; 3 Steno Diabetes Center North Jutland Aalborg Øst Denmark; 4 Department of Clinical Medicine Aalborg University Aalborg Øst Denmark; 5 Department of Endocrinology Aalborg University Hospital Aalborg Øst Denmark

**Keywords:** diabetes mellitus, glycemic control, HbA1c, telehealth

## Abstract

**Background:**

Telemedicine is often suggested as a promising approach to support patients with diabetes. However, the effectiveness of diabetes-related telemedicine interventions in regard to patient-related outcomes requires further evaluation. Previous systematic reviews describing the effectiveness of telemedicine in diabetes management focus on a specific type of telemedicine, a specific type of diabetes, specific comparators, or specific outcomes. Moreover, the rapid development within telemedicine emphasizes the need for a new review.

**Objective:**

The present review has a broad scope with an eye to performing an updated and exhaustive review within the field. The review aims to evaluate the effectiveness of existing telemedicine solutions versus any comparator without the use of telemedicine on diabetes-related outcomes among adult patients with diabetes.

**Methods:**

The review will consider studies that include adult subjects with a diagnosis of diabetes (type 1, 2, or gestational), studies that evaluate various types of telemedicine interventions, and randomized controlled trials comparing a telemedicine intervention to any control that does not include telemedicine. Peer-reviewed full-text papers in English, Norwegian, Danish, and Swedish will be considered. A thorough search will be performed in the PubMed, CINAHL, EMBASE, and Cochrane Library Central Register of Controlled Trials (CENTRAL) databases. Data extraction will include details about the populations, study methods, interventions, and outcomes of significance based on the review objective.

**Results:**

The results of the review are expected to provide an estimate of the treatment effect. The studies will be pooled via statistical meta-analysis and supplemented with narrative comparisons when necessary.

**Conclusions:**

The review is important as it will inform clinicians and investigators about the effect of various telemedicine solutions within the field of diabetes.

**International Registered Report Identifier (IRRID):**

DERR1-10.2196/22062

## Introduction

Diabetes poses a major health care problem worldwide. In 2017, an estimated 8.4% of the adult world population had diabetes, and this number is predicted to increase to approximately 9.9% (425 million) in 2045 due to an increase in unhealthy dietary habits, overweight, physical inactivity, and other risk factors [1,2]. Type 1 diabetes (T1D) and type 2 diabetes (T2D) represent approximately 5%-8% and 90%-95% of diabetes cases, respectively, and preexisting T1D, T2D, or gestational diabetes mellitus (GDM) occur in approximately 1 of 6 pregnancies [1,3-5].

T1D and T2D are associated with premature death and several complications such as cardiovascular disease, nephropathy, neuropathy, and retinopathy [6]. Preexisting diabetes during pregnancy and GDM is associated with both maternal and neonatal complications such as preeclampsia, miscarriages, stillbirth, birth complications, and congenital abnormalities [5,7]. Furthermore, women with GDM and their babies have a considerably higher risk of developing T2D later in life [7]. Optimal glycemic control is crucial for the prevention and control of diabetes-related complications [5,6]. However, sustaining optimal glycemic control in the context of diabetes is challenging given that diabetes is a demanding chronic disease that requires numerous daily self-management decisions and the performance of complex care activities. One example is the challenge in estimating the appropriate diabetes medication dosage to avoid hypo- and hyperglycemic events, which requires consideration of various influencing factors. Another example is adherence obstacles with regard to following the recommended guidelines [6,8].

Self-management support in the management of diabetes is a recognized approach to help people with diabetes navigate unavoidable decisions and activities related to the disease, and is known to be associated with improvements in health-related outcomes [8]. The American Association of Diabetes refers to diabetes self-management support as activities that provide educational, behavioral, psychological, or clinical assistance in “implementing and sustaining the behaviors needed to manage his or her condition on an ongoing basis” [9]. Hence, self-management support should be highly prioritized in the management of diabetes.

Telemedicine has been suggested as a promising but unproven approach to support people with diabetes in the control of their disease [10]. Telemedicine includes the use of telecommunication and information technology to deliver health care services, including monitoring, education, consultative services, and counseling tasks, at a distance. Telemedicine interventions embrace various constellations such as simple reminders via text messaging, video consultation, and transmission of patient data (eg, blood glucose, blood pressure, dietary and medication intake) with feedback from health care professionals via web portal interfaces or the telephone. As diabetes mainly needs to be managed outside health care facilities, telemedicine could be an obvious solution to provide self-management support to people with diabetes. Moreover, telemedicine could be relevant to those who have difficulties traveling to health care facilities due to disabilities or large distances [11,12]. A recent systematic review and meta-analysis concluded that the use of telehealth solutions among people with diabetes is a safe option for the delivery of self-management support [13]. However, the approach still needs to be sufficiently evaluated in regard to its effectiveness in terms of patient-related outcomes (eg, clinical, behavioral, and psychological) within the diabetes context. Given this situation, a comparison and review of the effectiveness of different types of telemedicine interventions is highly relevant.

Previous systematic reviews describing the effectiveness of telemedicine for the management of diabetes exist [10,14-24]. However, these reviews are limited to specific types of telemedicine (eg, telemonitoring) [14,17-22], a specific diabetes type [15,23,24], specific outcomes (eg, hemoglobin A1c) [10,16], or specific comparators (typically usual care) [10,22]. In T2D, a previous systematic review observed heterogeneity among the included telemedicine studies, which may partly be explained by the variations in the types of telemedicine used [8]. This observation underlines the need for a review including a specific analysis considering the different types of telemedicine. The call for a new systematic review and meta-analysis of telemedicine solutions among people living with diabetes is further indicated due to the rapid development in the field of telemedicine. Hence, the expectation is that a large number of additional studies have been published since the performance of the previous reviews. Moreover, the possibility of integrating more telemedicine solutions increases in relevance owing to the increasing focus on the efficiency of health care resources [25].

The above-mentioned considerations suggest the value of a new and extensive review in the field. Therefore, the objective of this systematic review and meta-analysis will be to evaluate the effectiveness of existing telemedicine solutions versus any comparator without the use of telemedicine on diabetes-related outcomes among adult patients with diabetes. Telemedicine is defined as telecommunication and information technology that delivers health care services at a distance [11,12].

## Methods

### Study Design

The review will be conducted and reported according to the PRISMA (Preferred Reporting Items for Systematic Reviews and Meta-analyses) guidelines [26], and a search protocol registered April 21, 2020 on PROSPERO (CRD42020123565) will form the basis of the review process.

### Review Question

The question of this systematic review and meta-analysis is as follows: How effective are telemedicine solutions versus any comparator without the use of telemedicine in regard to diabetes-related outcomes among adult patients with diabetes?

As the question indicates, the review has a very broad scope. Hence, the review considers (1) studies related to T1D, T2D, and GDM; (2) all patient-related outcomes (physiological/clinical, psychological, and behavioral); and (3) any comparator that does not include telemedicine.

### Inclusion Criteria

#### Participants

This systematic review and meta-analysis will consider studies that included adults ≥18 years of age who were diagnosed with T1D, T2D, or GDM. Studies that focused on pregnant women with preexisting T1D or T2D will also be considered. Studies reporting mixed disease populations (eg, diabetes and heart disease), mixed diabetes types, or mixed age groups (children and adults) will be included if the data for the population with diabetes or adult age group are reported separately in a transparent subgroup analysis. Studies will be excluded if they only include subjects at risk of diabetes or individuals with prediabetes.

#### Interventions

This systematic review and meta-analysis will consider studies that evaluate telemedicine interventions as a substitute or alternative to usual practice (ie, interventions with remote feedback/communication between either the patient and health care professional[s] or between the patient and trained peer[s]). Furthermore, wholly automatic telemedicine interventions will be considered. The telemedicine interventions may include various technologies such as telephone, smartphone, mobile phone, fax, text messaging, tablet, personal digital assistant, computer, and monitoring equipment (eg, glucometer, weight scale, pedometer, or sphygmomanometer) [11,12].

#### Comparators

This systematic review and meta-analysis will consider studies that compare the intervention to usual care or an alternative intervention without telemedicine. Both parallel and crossover designs will be considered.

#### Outcomes

This systematic review and meta-analysis will consider studies that report on any patient diabetes-related outcome(s) (ie, any physiological, psychological, or behavioral outcome). The primary outcome for T1D and T2D will be glycemic control, measured as a change in glycated hemoglobin (%). The primary outcome for pregnancy-related diabetes will be birth weight.

#### Study Types

This systematic review and meta-analysis will only consider randomized clinical trials (RCTs) with both a parallel and crossover design. Furthermore, studies will be included if the researchers consider the study to have adopted an RCT design based on the methodological approach of the individual study even if is the paper states that it used another design (eg, a quasiexperimental study design). Studies published in English, Danish, Norwegian, and Swedish as peer-reviewed full-text papers will be included. All studies published before the submission of the paper will be included, as both older and newer interventions are considered to bring value to the study (an updated search will be performed prior to submission).

### Search Strategy

The search strategy aims to locate both published and unpublished studies. An initial limited search of the PubMed and CINAHL databases will be undertaken to identify articles on the topic. The text words contained in the titles and abstracts of relevant articles and the index terms used to describe the articles will be used to perform a full search using PubMed, EMBASE, CINAHL, and the Cochrane Library Central Register of Controlled Trials (CENTRAL) databases. The full search history in PubMed is provided in Multimedia Appendix 1. The search strategy, including all identified keywords and index terms, will be adapted for each included information source. Search terms will include different synonyms, near-synonyms, spellings, and acronyms for each identified keyword and index term. Moreover, different search functions such as thesaurus, Boolean operators, truncation, abstract/title/keywords, phrase, free text, and advanced search will be applied to focus and structure the search.

To prevent selection bias, a follow-up search will be performed in each of the four databases prior to manuscript submission.

Citation searches and manual searches of the reference lists of relevant systematic reviews and of all studies selected for critical appraisal will be performed to identify additional studies.

### Information Sources

The databases to be searched are PubMed (no limitation of search years), EMBASE (no limitation of search years), CENTRAL (no limitation of search years), and CINAHL (no limitation of search years). Two review authors will perform the database searches in collaboration with a research librarian.

Citation searches will be performed in SCOPUS, Web of Science, and through Google Scholar. ClinicalTrials will be searched to identify unpublished studies.

Authors of identified articles will be contacted if an article is not possible to access or if any question occurs during the selection or extraction processes.

### Study Selection

Following the search, all identified citations will be collated and uploaded into RefWorks (Refworks, RefWorks-COS, ProQuest RefWorks 2.0, 2010), where the functions *Exact duplicates* and *Close duplicates* will be used for duplicate removal. Titles and abstracts will then be screened by two independent reviewers for assessment against the inclusion criteria of the review. Potentially relevant studies will be retrieved in full and assessed in detail by two independent reviewers against the inclusion criteria. Records on which the reviewers agree will be included in the systematic review. Any disagreement that arises between the reviewers at each stage of the study selection process will be resolved through discussion between the two reviewers or by including other reviewers in the decision-making process.

Reasons for exclusion of full-text studies that do not meet the inclusion criteria will be recorded and reported in the systematic review and meta-analysis. The results of the search will be reported in full in the final systematic review and meta-analysis, and presented in a PRISMA flow chart as shown in Multimedia Appendix 2 [26]*.*

### Assessment of Methodological Quality

The risk of bias assessment of eligible studies will be performed by two or more independent reviewers using the revised Cochrane risk-of-bias tool [27].

Authors of papers will be contacted to request missing or additional data for clarification when required. Any disagreements that arise between the reviewers will be resolved through discussion or with a third or additional reviewer(s). The results of the risk-of-bias assessment of the eligible studies will be reported in a risk-of-bias chart using the revised Cochrane risk-of-bias tool [27].

All studies, regardless of the results of their methodological quality, will undergo data extraction and synthesis (where possible).

### Data Extraction

Data will be extracted from the studies included in the review by three independent reviewers using tables in Microsoft Excel 2016. The data extracted will include specific details about the populations, study methods, interventions, and outcomes of significance based on the review objective. More specifically, three categories are considered relevant: (1) baseline characteristics of the study populations, (2) trial characteristics and key results, and (3) characteristics of the telemedicine interventions.

Any disagreements that arise between the reviewers will be resolved through discussion or with a third or additional reviewer(s).

### Data Synthesis

All included studies will be pooled via a statistical meta-analysis to provide an overall estimate of the treatment effect. Meta-analyses will be performed with the RevMan VX.X (Copenhagen: The Nordic Cochrane Centre, Cochrane) software tool for T1D and GDM, and with Stata 14 software (StataCorp 2015. Stata Statistical Software: Release 14. College Station, TX: StataCorp LP) for T2D. Effect sizes will be expressed as relative risks for dichotomous data. For continuous outcomes, effect sizes will be expressed as the mean (SD) change in outcomes from baseline to follow up for both groups, and, if not available, the absolute mean (SD) at follow up for both groups will be used. From this, the mean difference between the intervention and control group (or standardized mean difference) and confidence intervals can be calculated for each study. Heterogeneity will be assessed qualitatively by comparing the characteristics of the studies and statistically using I^2^ tests. Statistical analyses will be performed using a fixed-effects model except if heterogeneity is deemed as being present (I^2^>50%), in which case a random-effects model will be used. Subgroup analyses on types of telemedicine will be conducted when appropriate. Where statistical pooling is not possible, the findings will be presented in narrative form, including tables and figures to aid in data presentation when appropriate. A funnel plot will be generated to assess publication bias if there are 10 or more studies included in a meta-analysis. Statistical tests for funnel plot asymmetry (Egger test, Begg test, Harbord test) will be performed when appropriate.

### Data Reporting

Three systematic reviews and meta-analyses will be conducted to obtain the results: one report on studies related to diabetes during pregnancy, one report on T1D studies (not related to pregnancy), and one report on T2D studies (not related to pregnancy). This subdivision is considered relevant to ensure a clear and easily accessible report of the results. The flowchart of the search will be reported in each of the reviews as it appears in Multimedia Appendix 2.

### Assessing Certainty in the Findings

The Grading of Recommendations, Assessment, Development and Evaluation (GRADE) approach for grading the certainty of evidence will be used, and a Summary of Findings (SoF) will be created using GRADEPro GDT 2015 (McMaster University, ON, Canada; developed by Evidence Prime, Inc) [28]. The SoF will present the following information where appropriate: absolute risks for the treatment and control groups; estimates of relative risk; and a ranking of the quality of the evidence based on the risk of bias, directness, heterogeneity, precision, and risk of publication bias of the review results. The outcomes reported in the SoF will be hemoglobin A1c (%) for T1D and T2D and birth weight for pregnancy-related diabetes.

## Results

The systematic review is ongoing. A preliminary search in PubMed resulted in retrieval of 4813 records (Multimedia Appendix 1). The results of the review are expected to provide an estimate of the treatment effect. The studies will be pooled via statistical meta-analysis and supplemented with narrative comparisons when necessary. The results will be submitted for publication and peer review.

## Discussion

The review is expected to have strengths as well as limitations. On the one hand, the search of literature will be very inclusive and exhaustive, which is a strength. On the other hand, the review is expected to be limited by the lack of homogeneity between the included telemedicine studies, as telemedicine studies tend to differ in intervention type, inclusion criteria, technology, and other aspects. The comparison of studies is expected to be complicated due to this divergence.

## Appendix

Multimedia Appendix 1 Search strategy.

Multimedia Appendix 2 Study selection flowchart.

## Abbreviations

CENTRAL: Central Register of Controlled Trials
GDM: gestational diabetes mellitus
GRADE: Grading of Recommendations, Assessment, Development and Evaluation
PRISMA: Preferred Reporting Items for Systematic reviews and Meta-analyses
RCT: randomized clinical trial
SoF: Summary of Findings
T1D: type 1 diabetes
T2D: type 2 diabetes
