# Supplementary material for: Effectiveness of Telemedicine Solutions for the Management of Patients With Diabetes: Protocol for a Systematic Review and Meta-Analysis
Source: JMIR Res Protoc. 2020 Nov 3;9(11):e22062. doi: 10.2196/22062 (PMC7671833; doi:10.2196/22062)
Supplement: Multimedia Appendix 1 [file resprot_v9i11e22062_app1.docx]

**Multimedia Appendix 1:** Search strategy

*PubMed* (Date of last search: 21/09/2018)

| **Search** | **Query** | **Records retrieved** |
| --- | --- | --- |
| **Block 1 (Intervention - telemedicine)** | | |
| #1 | Telecommunications [MeSH] | 81773 |
| #2 | Telenursing [MeSH] | 189 |
| #3 | "Computer Communication Networks"[MeSH] | 83423 |
| #4 | (Telemedicin*[Title/Abstract] OR tele medicin*[Title/Abstract] OR telenurs*[Title/Abstract] OR tele nurs*[Title/Abstract] OR teleassist*[Title/Abstract] OR tele assist*[Title/Abstract] OR telebase*[Title/Abstract] OR tele base*[Title/Abstract] OR teleconsult*[Title/Abstract] OR tele consult*[Title/Abstract] OR telecouns*[Title/Abstract] OR tele couns*[Title/Abstract] OR telederm*[Title/Abstract] OR tele derm*[Title/Abstract] OR telediagnos*[Title/Abstract] OR tele diagnos*[Title/Abstract] OR telefollow*[Title/Abstract] OR tele follow*[Title/Abstract] OR teleguid*[Title/Abstract] OR tele guid*[Title/Abstract] OR telehealth*[Title/Abstract] OR tele health*[Title/Abstract] OR telehome*[Title/Abstract] OR tele home*[Title/Abstract] OR teleintervention*[Title/Abstract] OR tele intervention*[Title/Abstract] OR telemanag*[Title/Abstract] OR tele manag*[Title/Abstract] OR telemed*[Title/Abstract] OR tele med*[Title/Abstract] OR telemonitor*[Title/Abstract] OR tele monitor*[Title/Abstract] OR teleophthalmo*[Title/Abstract] OR tele ophthalmo*[Title/Abstract] OR telepatholog*[Title/Abstract] OR tele patholog*[Title/Abstract] OR teleprocedu*[Title/Abstract] OR tele procedu*[Title/Abstract] OR telerefer*[Title/Abstract] OR tele refer*[Title/Abstract] OR telerehab*[Title/Abstract] OR tele rehab*[Title/Abstract] OR teletherap*[Title/Abstract] OR tele therap*[Title/Abstract] OR teletreat*[Title/Abstract] OR tele treat*[Title/Abstract] OR telecare[Title/Abstract] OR tele care[Title/Abstract] OR telematic*[Title/Abstract] OR tele matic*[Title/Abstract] OR telecommunica*[Title/Abstract] OR tele communica*[Title/Abstract] OR telehomecare[Title/Abstract] OR tele home care[Title/Abstract] OR telehome care[Title/Abstract] OR tele homecare[Title/Abstract] OR mobile health[Title/Abstract] OR selfmonitor*[Title/Abstract] OR self monitor*[Title/Abstract] OR ehealth[Title/Abstract] OR digital health[Title/Abstract] OR dhealth[Title/Abstract] OR home monitor*[Title/Abstract] OR electronic care[Title/Abstract] OR ecare[Title/Abstract] OR econsult*[Title/Abstract] OR electronic consult*[Title/Abstract] OR ediagnos*[Title/Abstract] OR electronic diag*[Title/Abstract] OR electronic health[Title/Abstract] OR emedicine[Title/Abstract] OR electronic medicine[Title/Abstract] OR enurs*[Title/Abstract] OR electronic nurs*[Title/Abstract] OR ephysic*[Title/Abstract] OR electronic physic*[Title/Abstract] OR etherap*[Title/Abstract] OR electronic therap*[Title/Abstract] OR electronic mail*[Title/Abstract] OR mhealth[Title/Abstract] OR iphone*[Title/Abstract] OR smartphone*[Title/Abstract] OR PDA[Title/Abstract] OR Personal Digital Assistant*[Title/Abstract] OR phone*[Title/Abstract] OR tablet*[Title/Abstract] OR internet[Title/Abstract] OR compute*[Title/Abstract] OR app[Title/Abstract] OR application*[Title/Abstract] OR digital*[Title/Abstract] OR mobile*[Title/Abstract] OR cellphone*[Title/Abstract] OR telephone*[Title/Abstract] OR remote*[Title/Abstract] OR web base*[Title/Abstract] OR webbase*[Title/Abstract] OR cellular[Title/Abstract] OR landline[Title/Abstract] OR land line[Title/Abstract] OR blackberr*[Title/Abstract] OR black berr*[Title/Abstract] OR palmpilo*[Title/Abstract] OR palm pilot*[Title/Abstract] OR android[Title/Abstract] OR pocket PC*[Title/Abstract] OR radio*[Title/Abstract] OR walkee talkee*[Title/Abstract]) | [2783879](https://www.ncbi.nlm.nih.gov/pubmed/?cmd=HistorySearch&querykey=10) |
| #5 | #1 OR #2 OR #3 Or #4 | 2858553 |
| **Block 2 (Population- diabetics)** | | |
| #6 | “Diabetes Mellitus”[MeSH] | 388635 |
| #7 | (diabet*[Title/Abstract] OR DM[Title/Abstract] OR IDDM[Title/Abstract] OR NIDDM[Title/Abstract] OR MODY[Title/Abstract]) | 586571 |
| #8 | #6 OR #7 | 640577 |
| **Block 3 (Publication type - RCT)** | | |
| #9 | "Controlled Clinical Trials as Topic"[MeSH] | 125642 |
| #10 | "Controlled Clinical Trial" [Publication Type] | 556719 |
| #11 | ((((trial*[Title/Abstract] OR study[Title/Abstract] OR studies[Title/Abstract]))) AND random*[Title/Abstract]) AND control*[title/Abstract] | 412420 |
| #12 | #9 OR #10 OR #11 | 856466 |
| **Block 3 (combined block searches)** | | |
| #9 | #5 AND #8 AND #12 | 5037 |
| **Limited to** | | |
| #10 | #9Filters: Danish; English; Norwegian; Swedish | 4813 |
