# Supplementary figures and images for: Effectiveness of Telemedicine Solutions for the Management of Patients With Diabetes: Protocol for a Systematic Review and Meta-Analysis
Source: JMIR Res Protoc. 2020 Nov 3;9(11):e22062. doi: 10.2196/22062 (PMC7671833; doi:10.2196/22062)

**Multimedia Appendix 2:** Study selection process


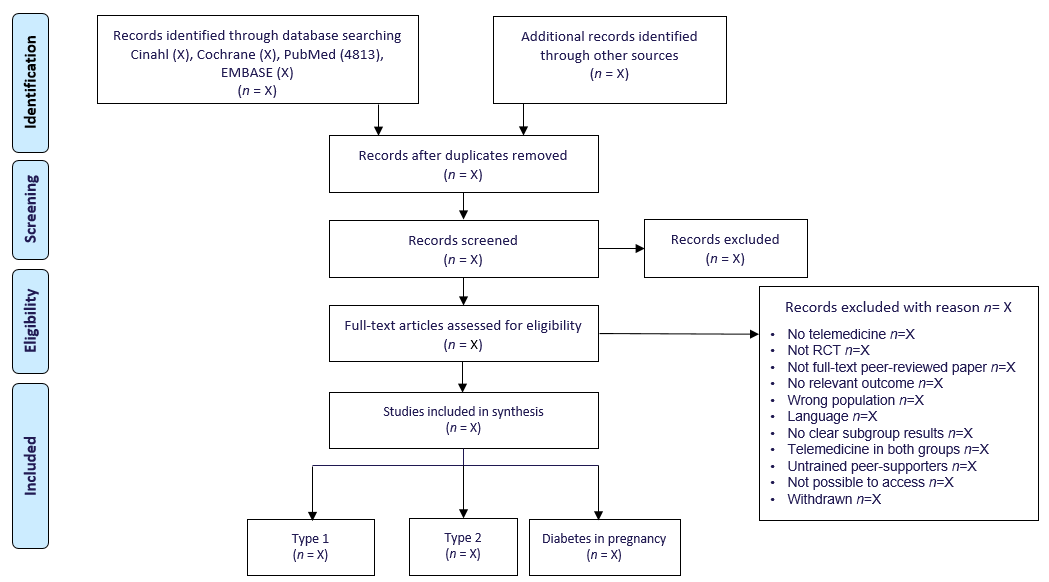

Supplement: Multimedia Appendix 2 [file resprot_v9i11e22062_app2.docx]
